# Risk assessment for oral urgent treatment in Primary Healthcare: a cross-sectional study

**DOI:** 10.1186/s12913-020-05859-2

**Published:** 2020-11-04

**Authors:** Danielle Viana Ribeiro Ramos, João Luiz Miraglia, Camila Nascimento Monteiro, Danielle Borchardt, Leonardo Tribis, Thais Paragis Sanchez, Daiana Bonfim, Danielle da Costa Palacio, Maria da Luz Rosário de Souza, Marília Jesus Batista de Brito Mota

**Affiliations:** 1 Hospital Israelita Albert Einstein, São Paulo, Brazil; 2 Faculdade de Odontologia de Piracicaba, Universidade Estadual de Campinas, Campinas, Brazil; 3 Faculdade de Medicina de Jundiaí, Campinas, Brazil

**Keywords:** Oral health, Emergencies, Risk assessment, Health services, Access, Primary healthcare

## Abstract

**Background:**

The World Health Organization has advocated for the integration of dental care into the primary healthcare (PHC) setting, including oral urgent treatment (OUT). However, the knowledge necessary for OUT implementation in this setting is still limited. Thus, this study aimed to describe the impact of the implementation of oral disease risk assessment tools for oral health management in PHC.

**Methods:**

This was a cross-sectional study that included individuals served by a single public PHC unit, with integrated oral healthcare teams, located in the south region of the city of São Paulo, Brazil, between April of 2015 and March of 2017. Data were collected from dental records. Three co-primary endpoints: same day treatment offered, first future appointment scheduled fulfilled, and treatment plan completed were compared before and after the implementation of oral disease risk assessment for OUT.

**Results:**

A total of 1214 individuals that sought OUT, 599 before and 615 after the implementation of oral disease risk assessment for OUT were included in the study. All three co-primary endpoints had significant changes after the implementation of oral disease risk assessment for OUT. Individuals were significantly more likely to be offered same day treatment after (39.9%; 95% CI:36.0–43.9%) than before (9.4%; 95% CI: 7.2–12.0%), to fulfill their first future appointment scheduled after (34.9%; 95% CI:31.1–38.8%) than before (20.7%; 95% CI: 17.5–24.2%), and to have their treatment plan completed after (14.3%; 95% CI:11.6–17.4%) than before (10.0%; 95% CI: 7.7–12.7%) the intervention.

**Conclusions:**

This study provided evidence of the positive impact oral disease risk assessment tools could have in the organization of OUT in PHC settings.

**Supplementary Information:**

The online version contains supplementary material available at 10.1186/s12913-020-05859-2.

## Background

Despite being largely preventable, oral diseases remain a global public health challenge affecting nearly half of the world’s population, particularly among low- and middle-income countries [1, 2]. Furthermore, dental diseases have an important economic impact on society [3], share common risk factors with other non-communicable diseases (e.g., free sugar consumption, tobacco use and wider social determinants of health), and might exacerbate the burden of other diseases [1, 4].

Although recognized as an integral part of overall health and wellbeing, oral health continues to be neglected [5]. In order to successfully address the global burden of these conditions, governments will need to implement an integrated preventive model into health systems, as opposed to the established westernized model of modern dentistry that is treatment focused and based mainly on high technology [1, 6].

The World Health Organization (WHO) has advocated for the integration of dental care into primary healthcare (PHC), enabling health promotion, prevention and favoring health equity. This approach encompasses different domains such as risk assessment, oral health evaluation, preventive interventions, communication, education, as well as interprofessional collaborative practices [7]. One of the three components of the Basic Package of Oral Health Care proposed by the WHO [8] is oral urgent treatment (OUT). Although many domains mentioned above could be used for OUT management, the relationship between service design, effective access and its impact on treatment management is yet to be assessed in a community setting [9]. Additionally, the use of oral disease risk assessment tools could help healthcare professionals to predict periodontitis progression and tooth loss in various populations [10, 11].

Thus, this study aimed to describe the impact of the implementation of oral disease risk assessment tools for oral health management in PHC. The hypothesis was that the implementation of such tools to organize spontaneous demand would allow oral health professionals to offer more equitable and effective services.

## Methods

### Ethics approval

This study was approved by the Research Ethics Committees of Hospital Israelita Albert Einstein (CAE: 84191318.2.0000.0071), the São Paulo Municipal Health Department (CAE: 92234418.2.3001.0086), and the School of Dentistry of Piracicaba, São Paulo, Brazil (CEP-FOP/UNICAMP), according to Resolution 466/12 of the Brazilian National Health Council. When a telephone number was available in the records of included participants, two telephone calls were made by the investigators in the attempt to obtain informed consent from included participants. The informed consent process included the request to use the participants’ dental records data in accordance with the study objectives and clear information reinforcing that refusal to participate in the study, or the withdrawal of consent at any moment, would not have any impact on the participants’ medical treatment or follow-up. The informed consent, obtained from the patients that accept to participate of the study, was written.

### Study design, population and setting

This was a cross-sectional study, conducted between April of 2015 and March of 2017, and included patients served by a single PHC unit located in the southern region of the city of São Paulo, Brazil. Three oral healthcare (OHC) teams served this PHC unit population, and each was composed of a dental surgeon, an oral health technician and an oral health assistant.

Although the state of São Paulo has the highest development index in the country [12], there is great variability among and within cities in the state. The city of São Paulo in particular has considerable, persistent and rising income disparities, and the region included in the study has areas of high levels of vulnerability [13].

The Brazilian health system is made up of a public-private mix with three interconnected subsectors: the public national health system (Sistema Único de Saúde or SUS), the private (for-profit and nonprofit), and the private health insurance subsectors, and individuals can use services in all three [14]. All publicly financed health services and most common medications are universally accessible and free of charge for all citizens, and since 1994 the Family Health Program (now called the Family Health Strategy, or FHS) has reorganized PHC in the public sector, with an estimated population coverage of 64% in 2016 [15, 16]. Since 2003, oral health policy moved from a secondary position to assuming a prominent position in the Brazilian government’s agenda. The National Oral Health Policy, called Smiling Brazil and published in 2004, reorganized primary OHC through the introduction of OHC teams into the FHS [17].

### Oral disease risk assessment instruments

In April 2016, three different oral disease risk assessment instruments (see Supplementary file 1) began to be used to organize OUT follow-up and treatment planning: a biological risk assessment [18], a risk assessment with colored risk categories [19], and a family health risk assessment (Coelho and Savassi’s scale) [20]. The risk assessment with colored risk categories is based on referred symptoms (hypersensitivity, periapical pain, periodontal pain, traumatic urgencies, hemorrhagic urgencies, stomatognathic apparatus emergencies, and mucosa urgencies), signs and the physical examination (Yellow: treatment priority; Green: same day treatment; Blue: non-urgent treatment/Future appointment scheduling), and was used to determine the initial approach towards patients seeking OUT. The family health risk assessment uses social, demographic and economic characteristics with the objective of evaluating health vulnerability (high, moderate or low), and was used in conjunction with the colored risk categories to determine the necessity of treatment priority. The biological risk assessment was based on physical examination findings to classify the risk of dental caries (high, moderate or low), periodontal disease (high, moderate or low) and soft tissue disease (yes or no). It was also used in conjunction with the family health risk assessment during the first scheduled appointment to help organize treatment planning.

In order to evaluate the impact of the implementation of oral disease risk assessment for OUT three co-primary endpoints were defined for each patient seeking care: a) same day treatment offered; b) first future appointment scheduled fulfilled; c) treatment plan completed.

### Data acquisition and statistical analysis

All individuals seeking OUT from April of 2015 to March of 2017, 1 year before until 1 year after the implementation of oral disease risk assessment for OUT, were eligible to be included in the study. Data from included individuals were extracted from paper-based dental records and entered directly into an electronic spreadsheet, by a single investigator at a single time. Missing data was excluded from the analyses.

The OUT visit percentage of the total of OHC visits, with its respective exact binomial 95% confidence interval (CI), was obtained for each study period. The chi-square test was used to compare percentages between periods.

Patients’ ages were categorized in five brackets (0–11, 12–19, 20–29, 30–59 and ≥ 60 years), while clinical complaints and diagnoses were categorized as described in Table 1. Risk of dental caries was categorized as high or moderate/low, periodontal disease as high or moderate and soft tissues disease as present or not, following guidelines from the municipal health department of São Paulo (see Table 5 in Supplementary file 1) [18].

**Table 1:** Characteristics of individuals seeking oral urgent treatment before and after the implementation of risk assessment

	Before implementation (***N*** = 598)	After implementation (***N*** = 615)	p*
	% (95% CI)		
**Age (years)**	*n* = 593	*n* = 600	0.307
0–11	21.0 (17.8–24.5)	23.0 (19.7–26.5)	
12–19	10.8 (8.4–13.5)	11.6 (9.2–14.5)	
20–29	16.0 (13.1–19.2)	19.0 (15.9–22.3)	
30–59	45.0 (41.1–49.2)	39.0 (35.2–43.1)	
≥ 60	7.1 (5.1–9.4)	7.3 (5.4–9.7)	
**Sex**	*n* = 598	*n* = 615	0.758
Female	60.9 (56.7–64.7)	60.0 (55.9–63.8)	
Male	39.1 (35.3–43.3)	40.0 (36.2–44.1)	
**Clinical Complaint**	*n* = 598	*n* = 615	0.515
Pain	46.4 (42.4–50.5)	43.2 (39.2–47.2)	
Tooth fracture	30.7 (27.0–34.6)	33.1 (29.4–37.0)	
Gingivitis	6.8 (5.0–9.2)	5.5 (3.9–7.6)	
Shedding of deciduous teeth	6.5 (4.7–8.8)	6.5 (4.7–8.7)	
Swelling	4.2 (2.7–6.1)	6.0 (4.3–8.2)	
Other	3.8 (2.4–5.7)	3.4 (2.1–5.2)	
Trauma	1.5 (0.7–2.8)	2.3 (1.2–3.8)	
**Diagnostic hypotheses**	*n* = 598	*n* = 615	< 0.001
Caries/pulp-related disease	80.5 (77.1–83.6)	70.1 (66.3–73.7)	< 0.001
Periodontal disease	9.0 (6.8–11.6)	8.5 (6.4–10.9)	0.730
Trauma	3.7 (2.3–5.5)	9.3 (7.1–11.8)	< 0.001
Eruption	2.7 (1.5–4.3)	5.4 (3.7–7.5)	0.017
Exfoliation	1.8 (0.9–3.3)	3.7 (2.4–5.6)	0.044
Other	2.3 (1.3–3.9)	3.1 (1.9–4.8)	0.420
**Caries risk**	*n* = 596	*n* = 614	< 0.001†
High	99.5 (98.5–99.9)	93.8 (91.6–95.6)	
Moderate/Low	0.5 (0.1–1.5)	6.2 (4.4–8.4)	
**Periodontal disease risk**		*n* = 614	
High		14.6 (11.9–17.7)	
Moderate		85.4 (82.3–88.1)	
**Soft tissue disease risk**		*n* = 614	
Yes		0.8 (0.3–1.9)	
No		99.2 (98.1–99.7)	
**Family risk**^**a**^		*n* = 612	
High		4.2 (2.8–6.2)	
Moderate		8.5 (6.4–11.0)	
Low		87.3 (84.4–89.8)	
**Color Risk**^**b**^		*n* = 612	
Yellow		22.5 (19.2–26.0)	
Green		42.2 (38.2–46.2)	
Blue		35.3 (31.6–39.3)	
**Same day treatment offered**	*n* = 597	*n* = 615	< 0.001
Yes	9.4 (7.2–12.0)	39.9 (36.0–43.9)	
No	90.6 (88.0–92.8)	60.1 (56.1–64.0)	
**Future first appointment scheduled fulfilled**	*n* = 598	*n* = 606	< 0.001
Yes	20.7 (17.5–24.2)	34.9 (31.1–38.8)	
No	79.3 (75.8–82.5)	63.6 (59.7–67.4)	
**Treatment plan completed**	*n* = 598	*n* = 606	0.022
Yes	10.0 (7.7–12.7)	14.3 (11.6–17.4)	
No	90.0 (87.3–92.3)	85.7 (82.6–88.4)	

Abbreviation: *CI* Confidence interval

*Chi-square test

†Fisher’s exact test

^a^Coelho and Savassi’s scale

^b^Yellow = treatment priority; Green = same day treatment; Blue = non urgent treatment/Future appointment scheduling

Co-primary endpoints were compared before and after the implementation of oral disease risk assessment. Percentages with respective exact binomial 95% CIs were obtained for every variable before and after the implementation of oral disease risk assessment for OUT. Chi-square test and Fisher’s exact test was applied to compare percentages between time periods.

Logistic regression was used to evaluate the impact of the implementation of oral disease risk assessment for OUT for each of the three co-primary endpoints. The initial full model was adjusted for age (continuous), sex, diagnosis, and dental caries risk, with no interaction terms included. A backward elimination variable selection algorithm with a significance level criterion of 0.2 was used to select the final models [21].

Data for periodontal disease risk and soft tissue disease risk before the implementation of oral disease risk assessment for OUT were largely missing.

Logistic regression was also used to evaluate the association between the three co-primary endpoints and clinical profile (clinical complaint, diagnosis, dental caries risk, periodontal disease risk and soft tissue disease risk) before and after implementation.

The analyses were conducted with Stata 10.1 (StataCorp. 2007. Stata Statistical Software: Release 10. College Station, TX: StataCorp LP) and used a significance level of 0.05.

## Results

PHC routinely-collected data from 18,595 individuals was assessed, with a total of 1215 individuals who sought OUT during both study periods, 598 before and 615 after the implementation of oral disease risk assessment. Phone numbers were available for 133 individuals of this total, with 84 being successfully reached and only one refusing consent, resulting in a study population of 1214 individuals, x before and y after the implementations of oral disease risk assessment. During the study period, 16.5% (95% CI: 15.3–17.7) of OUT visits were registered before and 20.8% (95% CI: 19.4–22.3) of OUT visits were registered after the implementation of oral disease risk assessment, a statistically significant difference (*p* < 0.001).

The distribution of the characteristics of individuals seeking OUT and the results for the three co-primary endpoints during both study periods is presented in Table 1.

Both periods included approximately 60% females, 40% were adults between 30 to 59 years of age, while approximately 45 and 30% of the clinical complaints were pain and tooth fracture, respectively. No significant differences were found between periods for these variables. Most individuals seeking OUT presented a high dental caries risk during both study periods, however this risk was significantly smaller after (93.8%; 95% CI: 91.6–95.6%) the implementation of oral disease risk assessment than before (99.5%; 95% CI: 98.5–99.9%). The most frequent diagnosis was dental caries/pulp-related diseases for both study periods, which followed the decrease seen in dental caries risk, being significantly less frequent after (70.1; 95% CI: 66.3–73.7%) the intervention than before (80.5%; 95% CI: 77.1–83.6%). Most individuals seeking OUT after the implementation of oral disease risk assessment presented a moderate risk of periodontal disease (85.4%; 95% CI: 82.3–88.1%), a low family risk (87.3%; 95% CI: 84.4–89.8%), no risk for soft tissue disease (99.2%; 95% CI: 98.1–99.7%) and were color risk classified as green (42.2%; 95% CI: 38.2–46.2).

All three co-primary endpoints had significant changes after the implementation of oral disease risk assessment for OUT, with the greatest improvement found for treatment offered on the same day, however low percentages of completed treatments were found for both study periods. Individuals were significantly more likely to be offered same day treatment after (39.9%; 95% CI:36.0–43.9%) than before (9.4%; 95% CI: 7.2–12.0%), to fulfill their first future appointment scheduled after (34.9%; 95% CI:31.1–38.8%) than before (20.7%; 95% CI: 17.5–24.2%), and to have their treatment plan completed after (14.3%; 95% CI:11.6–17.4%) than before (10.0%; 95% CI: 7.7–12.7%) the intervention.

Similar results were found by the adjusted analyses, with patients seeking OUT after the implementation of oral disease risk assessment 6.2 times more likely to be offered same day treatment (OR: 6.2; 95% CI: 4.4–8.6; *p* < 0.001; final model adjusted for sex, dental caries risk, and clinical complaints: tooth fracture, shedding of deciduous teeth and trauma), 2.3 times more likely to fulfill their first future appointment scheduled (OR: 2.3; 95% CI: 1.7–2.9; *p* < 0.001; final model adjusted for dental caries risk), and 1.6 times more likely to complete their planned treatment (OR: 1.6; 95% CI: 1.1–2.2; *p* = 0.013; final model adjusted for dental caries risk, and clinical complaints: gingivitis and tooth fracture) than before.

The associated factors to three co-primary endpoints adjusted by clinical patient profile (clinical complaint, diagnosis, dental caries, periodontal and soft risk) are described in Table 2.

**Table 2:** Association between outcomes and clinical patient profile. São Paulo, 2015–2017

	Same day treatment offered	First future appointment scheduled fulfilled	Treatment plan completed
**Clinical Complaint**^**a**^			
Pain	1	1	1
Broken tooth	1,16 (0,83-1,64)	1,23 (0,88-1,71)	1,15 (0,76-1,55)
Swelling	1,12 (0,59-2,25)	1,05 (0,54-2,03)	1,14 (0,49-2,68)
Gum inflammation	1,41 (0,77-2,57)	1,44 (0,79-2,62)	1,73 (0,84-3,68)
Trauma	**2,71 (1,05-6,93)**	**2,78 (1,11-7,04)**	0,51 (0,11-2,43)
Others	1,33 (0,610-2,89)	1,04 (0,44-2,47)	1,48 (0,51-2,69)
**Diagnostic Hypothesis Groups**^**b**^			
Caries/pulp-related disease	1	1	1
Periodontal disease	1,16 (0,69-1,92)	1,48 (0,59-3,85)	1,37 (0,67-2,83)
Trauma	1,02 (0,59-1,75)	0,94 (0,49-1,84)	1,11 (0,52-2,32)
Others	1,81 (0,81-4,02)	0,81 (0,54-1,47)	0,38 (0,79-1,82)
**Carie risk**			
No	1	1	1
Yes	1,01 (0,96-1,05)	1,01 (0,98-1,06)	0,97 (0,87-1,12)
Periodontal risk			
No	1	1	1
Yes	1,00 (0,98-1,01)	1,00 (0,99-1,01)	0,99 (0,98-1,01)
Soft tissues risk			
No	1	1	1
Yes	0,99 (0,98-1,01)	1,00 (0,99-1,08)	1,00 (0,98-1,02)

^a^Adjusted by Age, Sex, Diagnostic Hypothesis Groups, Carie Risk, Periodontal Risk, Soft Tissue risk

^b^Adjusted by Age, Sex, Clinical complaint, Carie Risk, Periodontal Risk, Soft Tissue risk

When comparing the patient’s profile according to implementation (before and after), although not statistically significant, there is a tendency to be more likely to be seen on the same day, first visit and have their treatment plan completed due to implementation. The trauma after the implementation was most likely to be served on the same day and future appointment scheduled fulfilled.

## Discussion

Before the implementation of oral disease risk assessment, oral health teams did not use any criteria to organize treatment demand, which was resolved with a first-come, first-served approach.

A recent review [22] identified some basic principles associated with improved access to healthcare services: matching supply to demand, immediate engagement of patient’s needs, patient preference on the timing and nature of care, need-tailored care, surge contingencies, and continuous assessment of changing circumstances. The use of risk assessment tools, especially for OUT, could facilitate the implementation of each one of these principles, enhance the effectiveness of primary OHC services, and have a broader impact, since timely access to urgent dental care decreases inappropriate attendance at general medical services and hospital emergency departments [9].

Furthermore, risk assessment tools could help to successfully integrate OHC into PHC. However, the multidimensional nature of oral diseases, including physical, psychological, emotional and social domains, demands different approaches such as the family health risk assessment used in this study. Family health vulnerability evaluation done by OHC teams could prompt actions from other PHC professionals promoting interprofessional collaborative practices, which have been shown to favor the successful integration of OHC into PHC [7].

This study found that the implementation of clinical and health vulnerability risk assessment for OUT was associated with an increased chance of individuals being offered same day treatment, fulfilling their first future appointment scheduled, and having their treatment plan completed. These results could have a direct effect on the continuity of care, which has been associated with better outcomes, higher satisfaction rates and more cost-effective care in the PHC setting [23].

The proportion of completed treatments was low during both study periods, but with a significant increase after the implementation of risk assessment for OUT. An increase of 30% was observed in treatments offered on the same day and 14% increase in fulfilled future first appointments scheduled. This confirms the utility of oral disease risk assessment tools to organize OUT and improve the access and effectiveness of primary OHC.

Dental caries risk was high among the study population, and a significant decrease in its frequency was found after the implementation of oral disease risk assessment. A similar significant decrease for dental caries/pulp-related diseases, the most frequent diagnosis made, was observed. A significant increase in the percentage of urgent visits from all OHC visits was identified after the implementation of oral disease risk assessment; however, this finding could be a consequence of increased access and a follow-up period not long enough to demonstrate a possible impact in the demand for OUT.

The association identified between periodontal disease and family health risk is in accordance with a previous study that evaluated parents of schoolchildren finding similar results [24].

More women sought OUT, which was expected since women are known to consult more often than men in PHC [25, 26]. The main complaint referred to by individuals in the study was pain, as observed elsewhere [26, 27].

Finally, in addition to have included only a single center limiting the external validity of its results, this study is also subject to the limitations of observational studies and of routinely-collected health data, with the possibility of the interference of multiple errors and biases (e.g., misclassification bias and underreporting) [28]. However, the use of routinely-collected health data presents the advantages of maximizing representativeness and generalizability, since data is collected under real-world circumstances, and of minimizing costs and effort.

## Conclusions

This study provided relevant evidence regarding the impact of oral disease risk assessment tools for the organization of OUT in the PHC setting. Hopefully, these results should encourage further evaluation of oral disease risk assessment tools in similar settings.

## Supplementary Information

**Additional file 1.** Oral disease risk assessment tools.

## Data Availability

The datasets used and/or analyzed during the current study are available from the corresponding author on reasonable request.

## Abbreviations

WHO: World Health Organization
PHC: Primary healthcare
OUT: Oral urgent treatment
OHC: Oral healthcare
CI: Confidence interval
