# Supplementary material for: Risk assessment for oral urgent treatment in Primary Healthcare: a cross-sectional study
Source: BMC Health Serv Res. 2020 Nov 4;20:1012. doi: 10.1186/s12913-020-05859-2 (PMC7643441; doi:10.1186/s12913-020-05859-2)
Supplement: Supplementary file 1 — Additional file 1. Oral disease risk assessment tools. [file 12913_2020_5859_MOESM1_ESM.docx]

**Supplementary file 1: Risk assessment instruments**

1. **Risk assessment with colored risk categories** (reference number 19 in the main article)

| **Table 1**. Yellow classification. | |
| --- | --- |
| **Patient complaint** | **Yellow - treatment priority** |
| Hypersensitivity | Intense, continuous and spontaneous pain. |
| Periapical pain | With submandibular swelling and likelihood of airway involvement. |
| Periodontal pain | Strong pain, otalgia dysphagia, lymphadenopathy, trismus and swollen mucosa. |
| Traumatic urgencies | Dental mobility, dislocation and fracture. |
| Hemorrhagic urgencies | Uncontrolled spontaneous bleeding associated with medications or systemic bleeding disorders. |
| Stomatognathic apparatus emergencies | Dislocation of the condylar process out of the mandibular cavity, painful symptoms and trismus. |
| Mucosa urgencies | Fever, malaise, irritability, headache, vesicles and extremely painful gums. |

| **Table 2**. Green classification. | |
| --- | --- |
| **Patient Complaint** | **Green - same day treatment** |
| Hypersensitivity | Provoked pain. |
| Periapical pain | Pain, sensitivity positive to percussion and negative to thermal stimuli. |
| Periodontal pain | Throbbing, localized pain, positive response to pulp vitality tests. |
| Traumatic urgencies | History of oral trauma but no acute symptoms. |
| Hemorrhagic urgencies | Bleeding is not spontaneous and controllable, with associated pain. |
| Mucosa urgencies | Lesion present for some time, suspected of malignancy. |

| **Table 3**. Blue classification. | |
| --- | --- |
| **Patient Complaint** | **Blue - non urgent treatment/Future appointment scheduling** |
| Hypersensitivity | History of pulp dentin pain, but no acute presentation at the moment. |
| Periapical pain | History of periapical pain, but no acute presentation at the moment. |
| Periodontal pain | No pain, but there are teeth with periodontal disease, vertical dislocation and function. |
| Traumatic Urgencies |  |
| Hemorrhagic urgencies | History of bleeding, but no acute symptoms at the moment. |
| Mucosa urgencies | History of oral mucosal injury but no acute symptoms. |

1. **Family risk assessment (Coelho and Savassi’s scale)**

| **Table 4.** Family risk assessment (Coelho and Savassi’s scale).* | |
| --- | --- |
| **Condition** | **Points scored** |
| Mobility restriction | 3 |
| Physical impairment | 3 |
| Mental impairment | 3 |
| Poor sanitation conditions | 3 |
| Malnutrition | 3 |
| Drug addiction | 2 |
| Unemployment | 2 |
| Illiteracy | 1 |
| >6 months of age | 1 |
| ≥70 years of age | 1 |
| Diagnosis of hypertension | 1 |
| Diagnosis of diabetes | 1 |
| Ratio for the household: number of people/number of rooms >1 | 3 |
| Ratio for the household: number of people/number of rooms =1 | 2 |
| Ratio for the household: number of people/number of rooms <1 | 0 |
| *Translated by the authors. The original scale was not validated in English. Reference number 20 in the main article. | |

| **Total score** | **Family risk** |
| --- | --- |
| 5–6 | Low |
| 7–8 | Moderate |
| >9 | High |

1. **Biological risk assessment for oral diseases** (reference number 18 in the main article)

**Caries risk**

| **Table 5.** Caries risk assessment. | | |
| --- | --- | --- |
| **Classification** | **Group** | **Individual situation** |
| Low Risk | A | No caries lesion, no plaque, no gingivitis and/or no active white spot. |
| Moderate Risk | B | History of restored tooth, no plaque, no gingivitis and/or no white spot active. |
|  | C | One or more cavities with chronic caries lesions, but without plaque, without gingivitis and/or without active white spot. |
| High Risk | D | Without caries lesion or presence of restored tooth, but with presence of plaque, gingivitis and / or active white spot. |
|  | E | One or more cavities in acute caries lesion. |
|  | F | Pain and/or abscess. |

**Periodontal disease**

Based on the modified Russell index, only the index teeth for each sextant is examined and ranked or, in the absence of the index element, the adjacent same sextant.

| 14 | 11 | 26 |
| --- | --- | --- |
| 46 | 31 | 34 |

The index takes the tooth unit into account and assigns each index tooth a code, according to the signs of periodontal disease present, being classified according to the criteria of Table 6. The individual was then classified by the code of his worst sextant.

| **Table 6.** Sextant classification according to risk criteria for periodontal disease. | | |
| --- | --- | --- |
| **Classification** | **Code** | **Criteria** |
| Low risk | 0 | Element with healthy periodontium. |
|  | X | No teeth in sextant |
| Moderate risk | 1 | Element with gingivitis. |
|  | 2 | Element with supragingival calculus. |
|  | B | Sequel of anterior periodontal disease. |
| High risk | 6 | Element with subgingival calculus (visible through gingival retraction) and with reversible mobility or without mobility. |
|  | 8 | Element with irreversible mobility and loss of function. |

**Soft tissue disease**

Observation of soft tissues at the time of screening follows the classification presented at Table 7.

| **Table 7.** Individual classification according to soft tissue risk criteria. | | |
| --- | --- | --- |
| **Classification** | **Code** | **Criteria** |
| Low risk | 0 | Normal soft tissues. |
| Moderate risk | 1 | Changes not suspected of malignancy, not covered in Code 2. |
| High risk | 2 | Changes with suspected malignancy: ulcers present for more than 15 days, with painful symptoms or not, raised edges or not; white and black lesions with ulcerated areas; red lesions with well-defined boundaries, suggesting erythroplasia; fast growing nodules with ulcerated areas. |
